# Ultrasonography of the metacarpophalangeal and proximal interphalangeal joints in rheumatoid arthritis: a comparison with magnetic resonance imaging, conventional radiography and clinical examination

**DOI:** 10.1186/ar1904

**Published:** 2006-03-06

**Authors:** Marcin Szkudlarek, Mette Klarlund, Eva Narvestad, Michel Court-Payen, Charlotte Strandberg, Karl E Jensen, Henrik S Thomsen, Mikkel Østergaard

**Affiliations:** 1 Department of Rheumatology, University of Copenhagen Hvidovre Hospital, Kettegård Allé 30, 2650 Hvidovre, Denmark; 2 Magnetic Resonance Research Centre, University of Copenhagen Hvidovre Hospital, Kettegård Allé 30, 2650 Hvidovre, Denmark; 3 Department of Radiology, University of Copenhagen Hvidovre Hospital, Kettegård Allé 30, 2650 Hvidovre, Denmark; 4 Department of Radiology, University of Copenhagen Herlev Hospital, Herlev Ringvej 75, 2730 Herlev, Denmark

## Abstract

Signs of inflammation and destruction in the finger joints are the principal features of rheumatoid arthritis (RA). There are few studies assessing the sensitivity and specificity of ultrasonography in detecting these signs. The objective of the present study was to investigate whether ultrasonography can provide information on signs of inflammation and destruction in RA finger joints that are not available with conventional radiography and clinical examination, and comparable to the information provided by magnetic resonance imaging (MRI). The second to fifth metacarpophalangeal and proximal interphalangeal joints of 40 RA patients and 20 control persons were assessed with ultrasonography, clinical examination, radiography and MRI. With MRI as the reference method, the sensitivity, specificity and accuracy of ultrasonography in detecting bone erosions in the finger joints were 0.59, 0.98 and 0.96, respectively; they were 0.42, 0.99 and 0.95 for radiography. The sensitivity, specificity and accuracy of ultrasonography, with signs of inflammation on T1-weighted MRI sequences as the reference method, were 0.70, 0.78 and 0.76, respectively; they were 0.40, 0.85 and 0.72 for the clinical examination. With MRI as the reference method, ultrasonography had higher sensitivity and accuracy in detecting signs of inflammation and destruction in RA finger joints than did clinical and radiographic examinations, without loss of specificity. This study shows that ultrasonography has the potential to improve assessment of patients with RA.

## Introduction

New aggressive and powerful treatments that permit fast and effective suppression of inflammation in rheumatoid arthritis (RA) demand sensitive and specific methods for detecting disease signs and monitoring disease activity. Finger joints are frequently the first to be involved in RA, and therefore methods of assessment of these joints are of particular importance at the onset of disease. The methods currently used, including clinical examination and conventional radiography, are not sensitive, especially in the evaluation of early stages of RA. In recent years magnetic resonance imaging (MRI) has been rigorously tested in patients with RA, and its value has been confirmed both in studies of large joints (for example, knee joints [1,2]) and in finger joints [3] compared with histological evaluation of biopsy specimens acquired at microarthroscopy. Thus far, because of the expensive equipment required and the need for highly qualified personnel, it has not become widely used as a joint assessment tool in RA. However, its benefits of high sensitivity and specificity in the evaluation of RA joints [4-6] make it a worthy surrogate 'gold standard' in settings where acquiring histological specimens is difficult (for example, finger joints).

Ultrasonography is an imaging technique that has attracted much interest in the field of rheumatology in recent years [7,8]. As a result of technological improvements and wide availability, ultrasonography has the potential to facilitate diagnosis of RA and improve the assessment of disease activity, and its use by rheumatologists may soon become routine. Few studies have compared ultrasonography with other imaging modalities with respect to their ability to detect signs of destruction and inflammation; furthermore, data are seldom gathered from homogenous populations and studies rarely include control persons. Despite of appearance in the literature of reports presenting the results of longitudinal studies of ultrasonographic assessment of RA, the more basic issues of agreement, sensitivity and specificity of ultrasonography in detecting RA pathology remain to be addressed.

We therefore planned a systematic study in order to investigate whether ultrasonography can provide information on RA finger joints that is not available with conventional radiography and clinical examination and comparable to the information provided by MRI.

## Materials and methods

### Patients

We examined a total of 158 second to fifth metacarpophalangeal (MCP) joints and 140 second to fifth proximal interphalangeal (PIP) joints of 40 patients with RA (fulfilling American College of Rheumatology 1987 criteria) and 80 second to fifth MCP joints and 80 second to fifth PIP joints of 20 healthy control persons. In the first part of the study we attempted to evaluate the wrists of RA patients, but after we had examined the first five patients the evaluation was omitted because of poor accessibility of most bone surfaces. The median age of the RA patients was 58 (range 23–79) years and that of the control persons was 52 (27–79) years. The female/male ratio was 4:1 both in the RA group and in the control group. The median disease duration in RA patients was 5 (range 0–20) years.

Twenty patients in the series had a disease duration in excess of 2 years (established disease). Their median age and disease duration were 64 (range 23–79) years and 8 (2–20) years, respectively. A further 20 patients had a disease duration of under 2 years (early disease). Their median and disease duration were 53 (range 23–72) years and 1 (0–1) year, respectively. All patients with established RA and 15 patients with early RA were being treated with disease-modifying antirheumatic drugs. The healthy control individuals had neither history of previous nor any current joint complaints.

The patients were recruited from two outpatient hospital-based arthritis clinics. The study was conducted in accordance with the Declaration of Helsinki and was approved by the local ethics committee. Signed informed consent was obtained from each participant. The inclusion criteria for RA patients were swelling or tenderness of at least three finger joints (MCP and/or PIP joints). The exclusion criteria were severe deformity of MCP or PIP joint and contraindications to MRI.

Ultrasonographic, clinical, laboratory and MRI examinations of each patient were conducted on the same day.

### Ultrasonography

**Figure 1:**
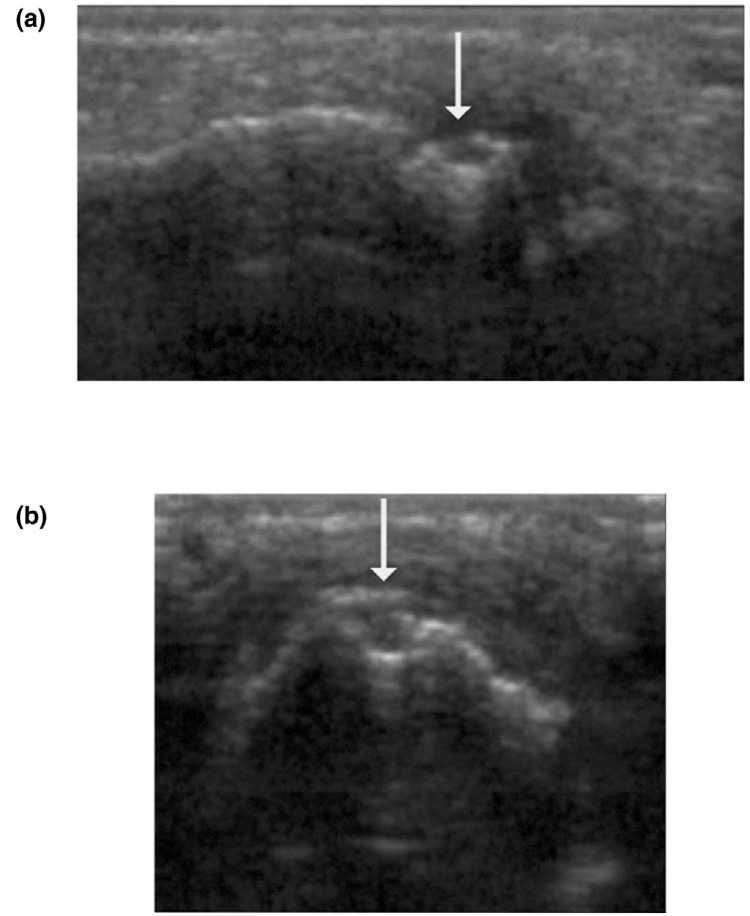
Signs of destruction on ultrasonography in the fourth proximal interphalangeal joint: early RA. MRI and conventional radiography revealed no signs of destruction in the joint. A bone erosion (arrow) is visualized with ultrasonography in **(a)** the longitudinal and **(b)** the transverse planes. MRI, magnetic resonance imaging; RA, rheumatoid arthritis.

**Figure 2:**
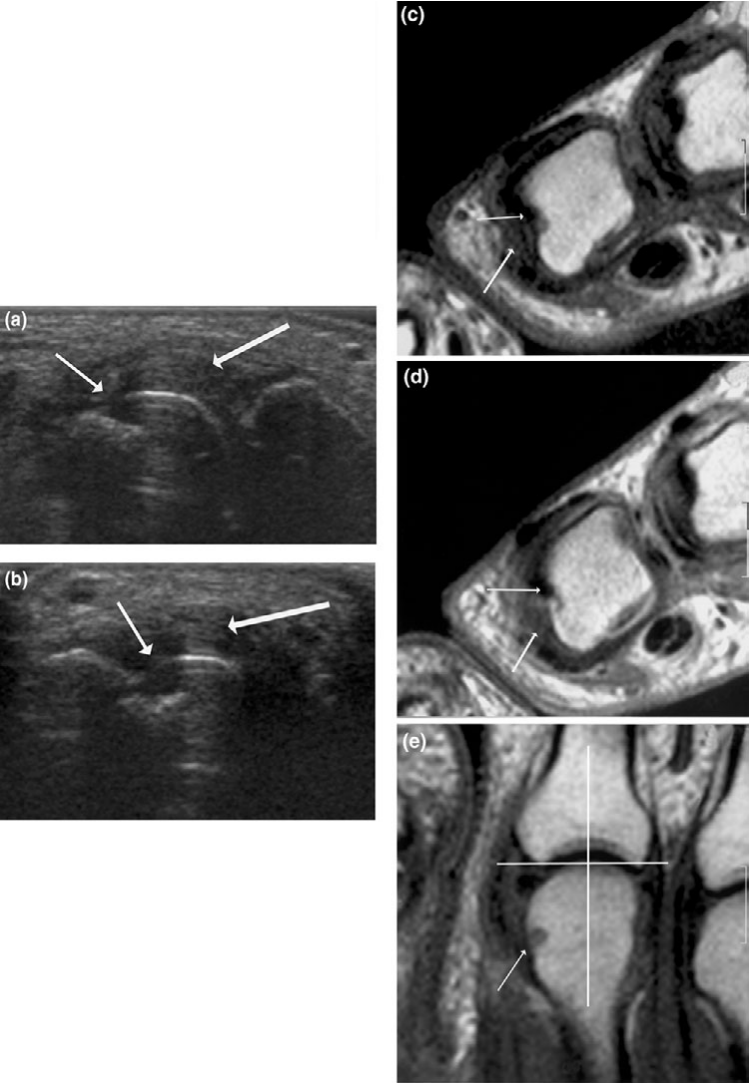
Signs of destruction and inflammation on ultrasonography and MRI in second metacarpophalangeal joint: established RA. Thin arrows indicate an erosive change; thick arrows indicate synovitis. Ultrasonography in the **(a)** longitudinal and **(b)** the transverse planes shows both signs of destruction (grade 2) and inflammation (grade 3). Axial T1-weighted magnetic resonance images were obtained **(c)** before and **(d)** after contrast administration (grade 3 synovitis). Additionally, a coronal T1-weighted magnetic resonance image **(e)** before contrast administration visualizes the same bone erosion as shown in panels c and d. The coronal magnetic resonance image of the second metacarpophalangeal joint (panel e) is additionally covered by a grid illustrating division of the assessed joints into quadrants: proximal radial, proximal ulnar, distal radial and distal ulnar. MRI, magnetic resonance imaging; RA, rheumatoid arthritis.

**Figure 3:**
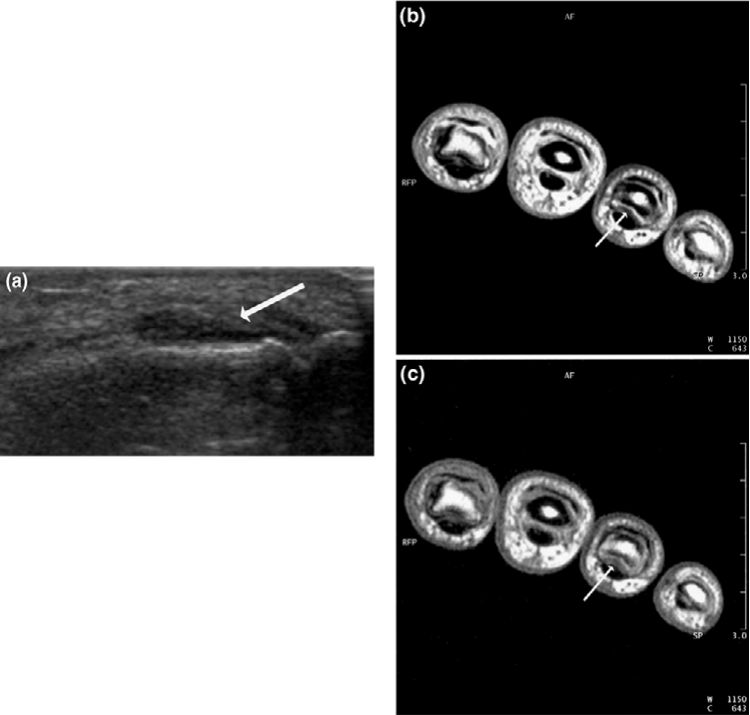
Signs of synovitis on ultrasonography and MRI in fourth proximal interphalangeal joint: early RA. Arrows indicate an area with synovitis. Ultrasonography in **(a)** the longitudinal plane from the dorsal aspect shows signs of synovitis (grade 4). Axial T1-weighted magnetic resonance images were obtained **(b)** before and **(c)** after contrast administration (grade 3 synovitis). MRI, magnetic resonance imaging; RA, rheumatoid arthritis.

Ultrasonography was performed using a General Electric LOGIQ 500 unit (General Electric, Solingen, Germany) using a 7–13 MHz linear array transducer. Ultrasonography was conducted in the accessible aspects of the second to fifth MCP joints and the second to fifth PIP joints of the dominant hand: the dorsal, radial and palmar aspects of the second MCP joint; the dorsal and palmar aspects of the third and fourth MCP joints; the dorsal, ulnar and palmar aspects the fifth MCP joint; and the dorsal, palmar, radial and ulnar aspects of all PIP joints. Ultrasonographic examination from the dorsal aspect was performed both in the neutral position and at about 70° of flexion. Each joint was assessed by quadrant for the presence or absence of bone erosions (Figures 1 and 2) and each joint was assessed for the presence or absence of signs of inflammation (joint effusion and synovitis; Figures 2 and 3).

The following definitions of ultrasonographic changes were employed: bone erosion = break in bone cortex in the area adjacent to the joint, visualized in two planes; joint effusion = compressible anechoic intracapsular area; and synovitis = uncompressible hypoechoic intracapsular area. The ultrasonographic changes were scored according to a semiquantitative scoring system (grades 0–3) introduced in an earlier report [9]. In relation to the original system, scoring of synovitis was widened to include grade 4, defined as a hypoechoic area bulging out of the joint and stretching over both bone diaphyses of the joint.

Ultrasonographic examinations were performed by two radiologists with expertise in musculoskeletal ultrasonography and a rheumatologist with training in the examination of the small joints of the extremities. Ultrasonography was performed without knowledge of the clinician's assessment or MRI data. The interobserver variation between one of the radiologists and the rheumatologist was presented in an earlier report [9].

### Clinical examination

Prior to ultrasonography, clinical disease activity (presence or absence of swelling and/or tenderness) in the MCP and PIP joints was evaluated in all patients by the consultant rheumatologist on duty. The number and localization of swollen and/or tender joints was determined.

### Conventional radiography

Radiography of the dominant hand was performed using standard postero-anterior and oblique (Nørgaard's) views within four weeks of the other examinations. The films were assessed by quadrant for the presence or absence of bone erosions in the second to fifth MCP joints and the second to fifth PIP joints by an experienced radiologist, who was unaware of the findings of the other examinations.

### Magnetic resonance imaging

Later in the day on which ultrasonography was performed, continuous axial and coronal pre-Gd-DTPA (gadolinium-diethylenetriamine penta-acetic acid) and post-Gd-DTPA T1-weighted spin-echo magnetic resonance sequences of the second to fifth MCP and second to fifth PIP joints of the dominant hand were performed. This MRI assessment employed a 1.0 T Siemens Impact MR unit (Siemens, Erlangen, Germany) equipped with a receive-only, wrap-around flex coil, and was conducted in the group with established disease, three patients with early disease and five control persons. The Gd-DTPA (0.1 mmol/kg body weight) was injected intravenously between repeated T1-weighted spin-echo magnetic resonance sequences. The patients and control persons were in the supine position with the hand in the coil along the femur. The parameters of the applied sequences were as follows for coronal sequences: repetition time (TR) 600 ms, echo time (TE) 15 ms, slice thickness (ST) 3 mm, field of view (FoV) 140 mm, and matrix 192 × 256. For axial sequences the parameters were as follows: TR 700 ms, TE 15 ms, ST 3 mm, FoV 120 mm, and matrix 192 × 256.

An extremity coil was used in 17 patients with early RA and 15 control persons. The use of different coils was necessary because technical problems meant that the wrap-around flex coil was unavailable for a lengthy period. The persons undergoing MRI were in supine position with the hand stretched above the head ('Superman' position). The parameters of the applied sequences for coronal sequences were as follows: TR 600 ms, TE 15 ms, ST 3 mm, FoV 145 mm, and matrix 192 × 256. For axial sequences the parameters were as follows: TR 600 ms, TE 15 ms, ST 3 mm, FoV 120 mm, and matrix 192 × 256.

The definitions of the applied MRI RA pathologies were in accordance with OMERACT recommendations [10].

The examinations were assessed by quadrant for the presence or absence of bone erosions (Figure 2) and by joint for the presence or absence of signs of inflammation (joint effusion and synovitis; Figures 2 and 3). Synovitis was scored according to the semiquantitative system (grades 0–4) introduced by Klarlund and coworkers [11]. The MRI observer was blinded to clinical and ultrasonographical data.

The numbers of finger joints assessed using ultrasonography/clinical examination and MRI were different (480 versus 433) because the MRI data for 47 joints were not available: 20 PIP joints were not visualized in the five patients in whom MRI of wrists and MCP joints was performed, and the MRIs of six MCP and 21 PIP joints were not assessable because the patients moved between pre- and post-contrast MRI sequences.

### Statistical analysis

The agreement between imaging methods and compared with clinical examination is reported as the overall agreement, defined as the proportion of exact agreements to the overall number of trials (expressed as a percentage). Furthermore, agreement was expressed as means of sensitivity and specificity. The correlation between ultrasonographic and MRI synovitis scores was estimated using calculations of intraclass correlation coefficients (ICCs; two-way mixed effects model, consistency definition).

## Results

### Signs of bone destruction

A total of 1,832 quadrants of second to fifth MCP joints (952 quadrants) and PIP joints (880 quadrants) from 40 RA patients and 20 healthy control individuals were examined using ultrasonography, MRI and radiography (Table 1).

In MCP joints, at least one modality detected bone erosions in 101 of 952 examined quadrants (11%). Agreement between all modalities on the presence of erosions was found in 29 out of 101 quadrants (29%), whereas ultrasonography and MRI agreed in 49 quadrants (49%). In 10 (11%) quadrants only ultrasonography and in 26 (26%) quadrants only MRI identified bone erosions. Half of the ultrasonographic erosions in RA patients that were not visualized by MRI were located in second and fifth MCP joints (7 out of 14), whereas MRI quadrants with erosions in RA patients not visualized with ultrasonography were located predominantly in third to fourth MCP joints (17 out of 23).

In PIP joints, at least one modality detected bone erosions in 27 of 880 quadrants (3%). Of these 27, only one quadrant (4%) was identified as erosive with all modalities. In 16 (59 %) quadrants only ultrasonography and in three (11 %) quadrants only MRI detected bone erosions. Ultrasonographic bone erosions, not visualized with other modalities, were distributed between all examined PIP joints, but most of them were located in the second and third PIP joints (15 out of 18). Radiography detected six (22%) quadrants with erosions in PIP joints that were not detected with other modalities.

Ten of the MRI quadrants with bone erosions in MCP joints were detected in healthy control persons (10 erosions in 238 MCP joints; frequency 4.2%), which is in contrast to none with ultrasonography and one with radiography. Ultrasonography and radiography detected no erosions in PIP joints of the healthy persons examined; one quadrant with erosions was found with MRI.

With MRI as the reference method, the sensitivity of ultrasonography in detecting bone erosions in the finger joints was 0.59, whereas it was 0.42 for radiography. The specificity of ultrasonography compared with MRI was 0.98, and for radiography compared with MRI it was 0.99. The accuracy of US (for instance the overall agreement between ultrasonography and MRI) for bone erosions was 0.96, and the accuracy of radiography was 0.95.

Erosive disease (defined as presence of at least one erosion in the examined finger joints) was found in 13 patients with radiography, in 20 with MRI, and in 20 with ultrasonography (15 simultaneously with MRI). Eleven patients with erosions on radiography were identified as having erosions with MRI and nine with ultrasonography. In the series of patients with early RA, ultrasonography visualized erosions in eight, MRI in six and radiography in three. In 20 control persons, MRI revealed erosions in seven; in one this was simultaneous with radiography.

The lowest grade (grade 1) of ultrasonographic erosive changes was visualized only in two cases out of 16 identified with MRI. Most of the definite (grade 2) ultrasonographic bone erosions were identified with MRI and some were identified with radiography. Almost all grade 3 ultrasonographic erosive changes were visualized with both MRI and radiography (Table 2). All of the most extensive ultrasonographic erosive changes (for instance grades 2 and 3) that were not detected with MRI or radiography were localized in PIP joints.

### Signs of inflammation

A total of 234 second to fifth MCP joints and 199 second to fifth PIP joints from 40 RA patients and 20 control persons were evaluated with ultrasonography, MRI and clinical examination. Agreement between ultrasonography and MRI regarding the presence or absence of synovitis was achieved in 76% (331/433) of the examined finger joints (Table 3). Furthermore, ultrasonography revealed signs of synovitis in 59 joints (14%) that were not detected with MRI, and MRI identified signs of synovitis in 43 joints (10%) that were not visualized with ultrasonography. Ultrasonography detected synovitis more often in patients with early RA than did MRI (88 versus 57 joints – a difference of 36%). The opposite was true in control persons, in whom MRI revealed synovitis more frequently than did ultrasonography (20 versus 5 joints – a difference of 75%). MRI did not detect joint effusion in any of the examined finger joints, whereas ultrasonography revealed joint effusion in 22 out of the 433 examined finger joints.

Signs of inflammation on ultrasonography (joint effusion and/or synovitis) were visualized in 194 out of 480 joints, whereas only 121 joints exhibited signs of inflammation at clinical assessment (swelling and/or tenderness; Table 4). Ultrasonographic and clinical findings agreed on the presence of signs of inflammation in 103 joints (21% of the 480 joints) and on the absence of signs of inflammation in 268 joints (56%). In 91 joints (19%), signs of inflammation (effusion or synovitis) on ultrasonography were found in clinically uninflamed joints, whereas no ultrasonographic signs of inflammation were observed in 18 joints (4%) in which the clinicians described swelling and/or tenderness. The overall agreement for both presence and absence of signs of inflammation between ultrasonography and clinical assessment was 77% (371 out of 480 examined finger joints).

The number of finger joints assessed with ultrasonography and MRI differed (480 versus 433) because the magnetic resonance data for 47 joints were not available: 20 PIP joints were not visualized in the five patients in whom MRI of wrists and MCP joints was performed, and MRIs of six MCP and 21 PIP joints were not assessable because the patients moved between pre- and post-contrast magnetic resonance sequences.

The sensitivity of ultrasonography, with signs of inflammation on T1-weighted MRI sequences as the reference, was 0.70 and the specificity was 0.78. The accuracy (for instance overall agreement between ultrasonography and MRI on signs of inflammation) was 0.76. The sensitivity of clinical examination, with signs of inflammation on T1-weighted MRI sequences as the reference, was 0.40 and the specificity was 0.85. The accuracy (for instance overall agreement between clinical examination and MRI on signs of inflammation) was 0.72.

Grading of synovitis with ultrasonography and MRI exhibited moderate-to-good correlations, as expressed by ICCs (two-way mixed effects model, consistency definition). ICCs for synovitis in the examined joints were as follows: second MCP 0.71, third MCP 0.61, fourth MCP 0.65, fifth MCP 0.58, second PIP 0.58, third PIP 0.58, fourth PIP 0.53, and fifth PIP 0.63. Results for exact agreement between scoring grades on ultrasonography and MRI are presented in Table 5.

The localization of joint inflammation was investigated, because signs of inflammation were assessed in all accessible aspects of the joints and registered separately. In MCP joints, of which 108 exhibited ultrasonographic signs of inflammation, these signs were present on both the dorsal and palmar aspect in 57 joints (52.7%), on the dorsal aspect only in 27 joints (25%), on the palmar aspect only in 19 joints (17.7%), and on the radial aspect only in five joints (4.6%). In PIP joints, of which 86 exhibited ultrasonographic signs of inflammation, these signs were present on both the dorsal and palmar aspect in 26 joints (30.2%), on the dorsal aspect only in 16 joints (18.6%), on the palmar aspect only in 37 joints (43%), and on the radial or ulnar aspect only in seven joints (8.1%).

## Discussion

In the present study we investigated the agreement between ultrasonography, radiography and clinical evaluation in the assessment of RA and healthy finger joints, with MRI as the reference method. It showed high agreement between ultrasonography and MRI in assessing RA bone erosions in finger joints. Using MRI as the reference method, ultrasonography exhibited markedly higher sensitivity in detecting RA bone erosions than did radiography, without loss of specificity.

In agreement with a study of RA MCP joints conducted by Wakefield and coworkers [12,13], we found that ultrasonography of those MCP joints with good accessibility by this modality (such as second and fifth) exhibited better correlations with MRI than did ultrasonography of joints only accessible in two planes (third and fourth). We found ultrasonographic bone erosions in many PIP joints in which MRI and radiography were unable to detect any destructive bone changes. This finding is probably explained by the use of 3 mm thick MRI slices, which must be considered suboptimal for the small PIP joints. In a heterogeneous group of patients with joint complaints, Backhaus and coworkers [14] did not find any advantage of ultrasonography over MRI in assessing bone destruction in PIP joints. This may be due to use of a 7.5 MHz transducer with a distance pad that is inferior to the high-frequency transducers employed in the present study. Furthermore, Backhaus and coworkers employed thinner MRI slices than were used in our study (1 mm versus 3 mm), favouring MRI over ultrasonography. Another possible reason for greater frequency of detection of erosions in PIP joints with ultrasonography may be its higher resolution in relation to MRI.

Preliminary data were reported by Alarcon and coworkers [15] and Lopez-Ben and colleagues [16] on the detection of bone erosions with ultrasonography in the second and fifth MCP joints of RA patients. They reported that ultrasonography had high accuracy, with MRI as the reference method, in the second and fifth MCP joints. In a group of patients with nonerosive RA on conventional radiography, Magnani and coworkers [17] visualized significantly more erosions in patients' MCP joints with ultrasonography than with MRI. Similar to our study, they used 3 mm thick MRI slices. Optimal technique would probably have improved the sensitivity of MRI.

Unlike in metatarsophalangeal joints [18], we found no ultrasonographic erosive changes in the examined finger joints of control persons; this is in contrast to MRI, which showed several single erosive changes in these joints. Erosive changes in control persons were detected with MRI with a frequency twice that reported in another study from our group (4.2% in the present study versus 2.2% in the study by Ejbjerg and coworkers [19]), but all except one were small. A possible reason for the MRI finding of erosions in the finger joints is that the visualized changes were subchondral cysts, which are not detected with ultrasonography because the employed ultrasound frequencies do not penetrate cortical bone. The less efficient/optimal blinding of the ultrasonographer as compared with the MRI evaluator might have caused bias toward finding fewer healthy control joints with erosions and synovitis by ultrasonography than by MRI.

The rate of detection of ultrasonographic destructive changes by MRI and radiography increased with the extent of erosion, as defined by its ultrasonographic grading. Correspondingly, the gradings of ultrasonographic inflammatory changes correlated with the volume-based MRI scoring of synovitis. Our results suggest that MRI and ultrasonography both allow assessment of abnormalities of the bone structures, and that performance differences are probably caused by technical aspects such as accessibility for ultrasonographic examination, high resolution of ultrasonographic assessment, or thickness of the MRI slices, rather than the physical principles of the examinations.

Ultrasonography had higher sensitivity for detecting signs of inflammation in the examined finger joints than did clinical examination, when MRI was considered the reference method, without considerable loss of specificity. Likewise, regarding the correlation of detection of synovitis between the methods, the moderate-to-good ICCs suggest that both ultrasonography and MRI were able to detect signs of inflammation. However, incomplete agreement between the methods suggested a margin of difference, probably due to ultrasonographic visualization of both 'active' and fibrotic pannus in the joints. The results are in agreement with those reported by Backhaus and coworkers [14], who showed greater frequency of detecting synovitis with ultrasonography than with MRI. A large proportion of 'disagreement', in which ultrasonography alone showed signs of synovitis, was found in patients with early RA. This suggests that fibrotic changes, which are probably less frequent in the early stages of the disease, are not the only changes identified by B-mode ultrasonography and not visualized on MRI. Current knowledge does not allow definite conclusions to be drawn regarding the cause of the discrepancy between ultrasonographic and MRI findings.

The difficulty associated with recognizing both 'active' and 'inactive' synovial tissue may be alleviated by the addition of Doppler ultrasonography. The growing number of reports comparing Doppler ultrasonography with MRI [20,21]. and histology of joints [22,23],. and describing the advantages of supporting ultrasonography with Doppler evaluation suggests that it will soon become a routine aspect of the joint assessment. However, many methodological and technical aspects of the use of Doppler ultrasonography remain to be clarified [24].

MRI did not permit visualization of joint effusion in RA finger joints, probably because of the minimal amount of fluid in the examined joints, whereas ultrasonography detected effusion in a considerable number of finger joints. This may be explained by the higher magnification of joints with ultrasonography than with MRI and the better resolution with ultrasonography. In our study, magnetic resonance images were read on hard-copy films. Evaluation on a computer screen, allowing magnification, would probably increase the sensitivity of MRI in detecting joint effusions. Additionally, MRI contrast diffusion into the joint cavity may contribute to making the detection of joint effusions with MRI more difficult [2,25]. In contrast to MRI, ultrasonography is a dynamic, real-time examination method, which permits evaluation of the findings in motion and under compression. The latter is a distinct feature of joint effusion on ultrasonography, which may explain the apparent advantage of this modality over MRI in detecting it.

In the present study the detection of joint effusion on ultrasonography did not improve its sensitivity in comparison with MRI on detecting signs of inflammation because it most often accompanied synovial thickening. However, in joints in which accessibility may be problematic, joint effusion could be used as indirect proof of an ongoing inflammatory process. Other researchers reported difficulty in differentiating between synovitis and joint effusion [14,26]. Standardization and precise definitions, as suggested in our earlier study [9], may be helpful in this respect.

Localization of signs of inflammation showed the dominance of the palmar aspect in PIP joints and a slight dominance of the dorsal aspect in MCP joints. In our opinion, the uneven distribution of signs of inflammation warrants examination of the joints from all possible aspects in order to avoid losing important information on the extent of inflammation [27].

With MRI as the reference method, ultrasonography almost doubled the sensitivity of assessing RA small joints for signs of inflammation compared with clinical assessment, without loss of specificity. The low sensitivity of clinical examination may account for the deterioration of RA patients despite clinically adequate control of the disease, as reported by Mulherin and coworkers [28]. Accordingly, a longitudinal study conducted by Backhaus and coworkers [29] showed progression of erosive changes with both ultrasonography and MRI, despite limited signs of clinical activity. The present study strongly suggests that clinical examination is far from optimal for assessing signs of inflammation in RA finger joints, and that the use of ultrasonography can considerably improve the detection of signs of synovial inflammation.

## Conclusion

Ultrasonography was shown to permit assessment of destructive and inflammatory changes in RA finger joints, with high agreement with MRI. Ultrasonography was more sensitive than plain film radiography in assessing bone destruction in the examined joints, and had equal specificity. B-mode ultrasonography was more sensitive than clinical examination in assessing signs of inflammation, with only a slight loss of specificity. The present study strongly encourages further studies of use of ultrasonography to assess RA finger joints.

## Abbreviations

FoV = field of view; Gd-DTPA = gadolinium-diethylenetriamine penta-acetic acid; ICC = intraclass correlation coefficient; MCP = metacarpophalangeal; MRI = magnetic resonance imaging; PIP = proximal interphalangeal; RA = rheumatoid arthritis; ST = slice thickness; TE = echo time; TR = repetition time.

## Competing interests

The authors declare that they have no competing interests.

## Authors' contributions

MS participated in the study development, performed the ultrasonographic evaluations, conducted the preliminary data evaluation and statistic analysis, and prepared the draft of the manuscript. MK took part in the study development, performed the MRI evaluation, and was involved in patient recruitment. EN performed the conventional radiographic evaluation. MC-P and CS performed ultrasonographic evaluations. KEJ took active part in study development and preliminary data interpretation. HST and MØ took part in the study development and gave substantial input into data evaluation and manuscript preparation. All authors read and approved the final manuscript.

## Figures and Tables

**Table 1:** Number of quadrants with bone erosions in finger joints, stratified by imaging modality and combinations thereof

Joint	Quadrants with erosions							Quadrants with no erosions on US, MRI or CR	Agreement		Sensitivity		Specificity	
				
	US + MRI + CR	US + MRI	MRI + RAD	US + CR	US only	MRI only	CR only		US versus MRI (%)	CR versus MRI (%)	US	CR	US	CR
MCP 2nd	10	10	2	1	2	7	3	205	225(94)	217(90)	0.64	0.41	0.98	0.98
Est.	9	8	2	1	1	2	2	55	72(90)	66(82)				
Early	1	2	0	0	1	2	0	74	77(96)	75(94)				
Control	0	0	0	0	0	3	1	76	76(95)	76(95)				

MCP 3rd	7	7	4	1	4	12	2	230	217(90)	214 (89)	0.47	0.37	0.97	0.98
Est.	6	6	4	1	3	6	2	52	64(80)	62 (78)				
Early	1	1	0	0	1	2	0	75	77(96)	76 (95)				
Control	0	0	0	0	0	4	0	76	76(95)	76 (95)				

MCP 4th	5	2	0	1	1	5	0	222	229 (97)	227 (96)	0.58	0.42	0.99	0.99
Est.	4	1	0	1	1	5	0	64	69 (91)	68 (89)				
Early	1	1	0	0	0	0	0	78	80 (100)	79 (99)				
Control	0	0	0	0	0	0	0	80	80 (100)	80 (100)				

MCP 5th	7	1	0	1	3	2	1	221	229 (97)	228 (97)	0.80	0.70	0.98	0.99
Est.	6	0	0	1	2	0	0	67	73 (96)	73 (96)				
Early	1	1	0	0	1	0	1	76	78 (97)	77 (96)				
Control	0	0	0	0	0	2	0	78	78 (97)	78 (97)				

PIP 2nd	0	0	0	0	6	1	1	212	212 (96)	212 (96)	-	-	0.97	0.99
Est.	0	0	0	0	5	0	1	54	54 (90)	54 (90)				
Early	0	0	0	0	1	0	0	79	79 (99)	79 (99)				
Control	0	0	0	0	0	1	0	79	79 (99)	79 (99)				

PIP 3rd	1	0	0	1	7	1	0	210	211 (96)	211 (96)	0.50	0.50	0.96	0.99
Est.	1	0	0	1	6	1	0	51	52 (87)	52 (87)				
Early	0	0	0	0	1	0	0	79	79 (99)	79 (99)				
Control	0	0	0	0	0	0	0	80	80 (100)	80 (100)				

PIP 4th	0	0	0	0	2	1	1	216	216 (98)	216(98)	-	-	0.99	0.99
Est.	0	0	0	0	2	0	1	57	57 (95)	57 (95)				
Early	0	0	0	0	0	1	0	79	79 (99)	79 (99)				
Control	0	0	0	0	0	0	0	80	80 (100)	80 (100)				

PIP 5th	0	0	0	0	1	0	4	215	215 (98)	215(98)	-	-	0.99	0.98
Est.	0	0	0	0	0	0	2	58	58 (97)	58(97)				
Early	0	0	0	0	1	0	2	77	77 (96)	77(96)				
Control	0	0	0	0	0	0	0	80	80 (100)	80 (100)				

Total	30	20	6	5	26	29	12	1,704	1,754 (96)	1,740 (95)	0.59	0.42	0.98	0.99
Est.	26	15	6	5	20	14	8	458	499 (90)	490 (89)				
Early	4	5	0	0	6	5	3	617	626 (98)	621 (97)				
Control	0	0	0	0	0	10	1	629	629 (98)	629 (98)				

The following numbers of joints were evaluated (1,832 in total): 240 MCP second, 240 MCP third, 236 MCP fourth, 236 MCP sixth, 220 PIP second, 220 PIP third, 220 PIP fourth, and 220 PIP fifth. All study participants included. CR, conventional radiography; early, early rheumatoid arthritis; Est., established rheumatoid arthritis; MCP, metacarpophalangeal joint; MRI, magnetic resonance imaging; PIP, proximal interphalangeal joint; US, ultrasonography.

**Table 2:** Detection of bone changes, visualized and scored with ultrasonography, by other imaging methods

		Bone erosions on MRI	Bone erosions on CR	No bone erosions on MRI and CR
US grades	Grade 1 (*n *= 16)	2	0	14
	Grade 2 (*n *= 55)	32	17	20
	Grade 3 (*n *= 26)	18	18	6

CR, conventional radiography; MRI, magnetic resonance imaging; US, ultrasonography.

**Table 3:** Numbers of joints with and without signs of synovitis in finger joints, stratified by imaging modality and combinations thereof

Joint	Joints with signs of synovitis			Joints with no signs of synovitis on US or MRI	Number of joints examined	Agreement: US versus MRI (%)
	US + MRI	US only	MRI only			
MCP 2nd	26	6	7	20	59	46 (78)
Est.	12	3	2	3	20	15 (75)
Early	14	2	0	3	19	17 (89)
Control	0	1	5	14	20	14 (70)

MCP 3rd	21	7	9	22	59	43 (73)
Est.	11	2	2	5	20	16 (80)
Early	10	4	2	3	19	13 (68)
Control	0	1	5	14	20	14 (70)

MCP 4th	11	7	3	37	58	48 (83)
Est.	6	2	2	9	19	15 (79)
Early	5	4	0	10	19	15 (79)
Control	0	1	1	18	20	18 (90)

MCP 5th	11	12	3	32	58	43 (74)
Est.	6	5	1	7	19	13 (68)
Early	5	7	0	7	19	12 (63)
Control	0	0	2	18	20	18 (90)

PIP 2nd	12	9	6	23	50	35 (70)
Est.	6	3	3	2	14	8 (57)
Early	6	5	1	5	17	11 (65)
Control	0	1	2	16	19	16 (84)

PIP 3rd	14	4	5	27	50	41 (82)
Est.	6	1	3	4	14	10 (71)
Early	7	3	0	7	17	14 (82)
Control	1	0	2	16	19	17 (89)

PIP 4th	10	5	4	31	50	41 (82)
Est.	5	1	3	5	14	10 (71)
Early	5	4	0	8	17	13 (76)
Control	0	0	1	18	19	18 (95)

PIP 5th	2	9	6	32	49	34 (69)
Est.	1	3	4	6	14	7 (50)
Early	1	6	1	8	16	9 (56)
Control	0	0	1	18	19	18 (95)

Total	107	59	43	224	433	331 (76)
Est.	53	20	20	41	134	94 (70)
Early	53	35	4	51	143	104 (73)
Control	1	4	19	132	156	133 (85)

All study participants included. early, early rheumatoid arthritis; Est., established rheumatoid arthritis; MCP, metacarpophalangeal joint; MRI, magnetic resonance imaging; PIP, proximal interphalangeal joint; US, ultrasonography.

**Table 4:** Signs of inflammation on ultrasonography versus clinical joint assessment in finger joints

Signs of inflammation	US + clinical assessment	US only	Clinical assessment only	Joints with no signs of inflammation on US or clinical assessment	Number of joints examined	Agreement: US versus clinical assessment (%)
MCP 2nd	23	12	3	22	60	45 (75)
MCP 3rd	20	10	5	25	60	45 (75)
MCP 4th	8	11	2	39	60	47 (78)
MCP 5th	8	16	2	34	60	42 (70)
PIP 2nd	13	13	1	33	60	46 (77)
PIP 3rd	13	11	3	33	60	46 (77)
PIP 4th	12	7	1	40	60	52 (87)
PIP 5th	6	11	1	42	60	48 (80)

Total	103	91	18	268	480	371 (77)

The number of examined joints is higher than in the other tables because ultrasonography and clinical examination were performed on all finger joints, whereas MRI data were not available in 47 joints. All study participants included. Ultrasonography detecting signs of synovitis and/or joint effusion. Clinical joint assessment detecting swelling and/or tenderness. MCP, metacarpophalangeal; PIP, proximal interphalangeal; US, ultrasonography.

**Table 5:** Comparison of scoring of synovitis with ultrasonography and MRI with their respective volume-based scales

		MRI grades				
		
		0	1	2	3	4
US grades	0	**75**	128	20	2	0
	1	13	**8**	19	1	1
	2	14	20	**12**	16	2
	3	9	12	13	**31**	21
	4	0	4	0	4	**8**

Values in the cells describe the numbers of joints, apart from those denoting score (first column for US and first row for MRI). Numbers in bold denote exact agreements between respective identical scores. MRI, magnetic resonance imaging; US, ultrasonography.
